# Effect on Adherence to Nicotine Replacement Therapy of Informing Smokers Their Dose Is Determined by Their Genotype: A Randomised Controlled Trial

**DOI:** 10.1371/journal.pone.0035249

**Published:** 2012-04-11

**Authors:** Theresa M. Marteau, Paul Aveyard, Marcus R. Munafò, A. Toby Prevost, Gareth J. Hollands, David Armstrong, Stephen Sutton, Chloe Hill, Elaine Johnstone, Ann Louise Kinmonth

**Affiliations:** 1 Psychology Department at Guy’s, Health Psychology Section, King’s College London, London, United Kingdom; 2 Primary Care Clinical Sciences, University of Birmingham, Birmingham, United Kingdom; 3 Department of Experimental Psychology, University of Bristol, Bristol, United Kingdom; 4 Department of Primary Care and Public Health Sciences, King’s College London, London, United Kingdom; 5 Department of Public Health and Primary Care, University of Cambridge, Cambridge, United Kingdom; 6 Department of Clinical Pharmacology, University of Oxford, Oxford, United Kingdom; Centre for Addiction and Mental Health, Canada

## Abstract

**Background:**

The behavioural impact of pharmacogenomics is untested. We tested two hypotheses concerning the behavioural impact of informing smokers their oral dose of NRT is tailored to analysis of DNA.

**Methods and Findings:**

We conducted an RCT with smokers in smoking cessation clinics (N = 633). In combination with NRT patch, participants were informed that their doses of oral NRT were based either on their mu-opioid receptor (*OPRM1*) genotype, or their nicotine dependence questionnaire score (phenotype). The proportion of prescribed NRT consumed in the first 28 days following quitting was not significantly different between groups: (68.5% of prescribed NRT consumed in genotype vs 63.6%, phenotype group, difference  =  5.0%, 95% CI −0.9,10.8, *p  = * 0.098). Motivation to make another quit attempt among those (n  =  331) not abstinent at six months was not significantly different between groups (*p*  =  0.23). Abstinence at 28 days was not different between groups (*p* = 0.67); at six months was greater in genotype than phenotype group (13.7% vs 7.9%, difference  =  5.8%, 95% CI 1.0,10.7, *p*  =  0.018).

**Conclusions:**

Informing smokers their oral dose of NRT was tailored to genotype not phenotype had a small, statistically non-significant effect on 28-day adherence to NRT. Among those still smoking at six months, there was no evidence that saying NRT was tailored to genotype adversely affected motivation to make another quit attempt. Higher abstinence rate at six months in the genotype arm requires investigation.

**Trial registration:**

Controlled-Trials.com ISRCTN14352545.

## Introduction

### Background

Pharmacogenomics has the potential to improve health outcomes in two key ways: first, by more effective prescribing, tailored to genotype; and second, by increasing motivation to take treatment. We present here the results of the first randomised controlled trial assessing the behavioural impact of informing smokers that their dose of medication is tailored to their genotype, as opposed to non-genetic information.

Both the initiation and maintenance of smoking have been reported as having high heritability [1], [2], [3]. There is also growing evidence that some of the variability in responsiveness to smoking cessation pharmacotherapy, such as nicotine replacement therapy (NRT) or bupropion, is explained by genotype [4], [5], [6]. Genetic tests are now available via the Internet (e.g. Respiragene (www.synergenz.com)) which claim to identify the optimal pharmacotherapy for an individual wishing to stop smoking or to motivate an individual to stop smoking but the impact of such testing has not been evaluated.

This trial uses testing for the Asp40 variant in the mu-opiod receptor (OPRM1) gene as its paradigm. During the design of the trial, the mu-opioid receptor (*OPRM1*) gene was a promising candidate which had been reported to be associated with smoking cessation [7]. In this original study, abstinence rates at follow-up among the group receiving the nicotine transdermal patch was ∼31% in those with one or more copies of the Asp40 variant (present in about 25% of the population), and ∼16% in those with two copies of the Asn40 variant. The rates among the group receiving the nicotine nasal spray were ∼15% and ∼13%, respectively. Therefore, Asp40 carriers appeared to show double the short term quit-rates when using a form of NRT with higher levels of replacement, compared with NRT that results in lower levels of replacement. However, it should be noted that the genotype × treatment interaction effect was not statistically significant in this study, and subsequent studies have failed to replicate this finding [8]. Nevertheless, for our purposes, whether individuals’ genotype influences smoking cessation is not directly relevant. The focus here is rather the behavioural impact of *communicating* to smokers that their medication has been tailored on a genetic basis. We therefore chose the *OPRM1* gene on the basis of evidence available at the time of trial design, to establish proof-of-principle of communicating genetic tailoring of medication.

There are high expectations that DNA-based risk information will motivate greater behaviour change than other types of biomarker risk information [9], [10], [11]. There are two possible mechanisms for such effects. First, information with high personal salience, such as DNA-based risk information, has a greater impact on attitude change than information with low personal salience [12]. Second, perceiving a health problem to have a genetic cause increases the perceived effectiveness of taking medication to deal with the problem [13]. This has been documented for depression, heart disease and stopping smoking [14], [15], [16], [17]. Given that perceived treatment effectiveness predicts treatment use [18], tailoring treatment on the basis of genetic testing has the potential to improve treatment outcomes by increasing adherence. Higher use of NRT is associated with a greater likelihood of smoking cessation [19], [20].

In contrast to these positive effects, informing patients that their prescription is tailored to their genotype may have negative effects through engendering a sense of fatalism, which is associated with perceiving a genetic cause to a health problem [21]. While perceived control seems unaffected by the communication of personalised genetic risk information [22], fatalism may be induced in those who fail to change their behaviour following such information. In the current context this could reduce motivation to make a future quit attempt amongst those who fail to quit.

#### Hypotheses

The trial tested two hypotheses:

*Hypothesis I*: Adherence to NRT is greater among smokers who are informed their oral dose of NRT is tailored to an analysis of DNA (genotype), rather than tailored to a nicotine dependence questionnaire score (phenotype).

*Hypothesis II*: Among smokers who fail to quit at six months, motivation to make another quit attempt is lower when informed that their oral dose of NRT was tailored to genotype rather than phenotype.

## Methods

### Ethical Approval

Ethical approval for the trial was granted (Hertfordshire 1 Research Ethics Committee, reference 06/Q0201/21) and the local primary care trusts in Birmingham and Bristol gave approval for the interventions. Additionally, the Medicine and Healthcare Products Regulatory Authority gave approval (MHRA ref: 24570/0002/001–0001).

The protocol for this trial and supporting CONSORT checklist are available as supporting information; see Checklist S1 and Protocol S1.

### Design Overview

An open label, parallel group, randomised controlled trial of NRT for smoking cessation in which participants were randomly allocated on a 1∶1 basis to one of two groups:

i. NRT oral dose tailored by DNA analysis, or
ii. NRT oral dose tailored by nicotine dependence score

We required a design which would result in a balance of gene variants and nicotine dependence across groups and would generate similar prescriptions of NRT in both groups, despite the communicated basis of prescriptions (genotype or phenotype) varying systematically by group. A design in which we would have randomised the rationale given for the prescription, but held the prescribed NRT constant (rather than it being tailored), was rejected as it would have meant deceiving participants about the true basis for their prescriptions.

### Setting and Participants

The trial took place in the British National Health Service (NHS) smoking cessation clinics in primary care. These provide a combination of weekly behavioural support and pharmacotherapy to assist smokers to quit. Participants were recruited from 29 primary care practices in two English cities, Birmingham and Bristol.Patients smoking an average of at least 10 cigarettes a day (including hand-rolled cigarettes), who wanted to quit and were 18 years or older, were eligible. Informed written consent was obtained from all participants involved in the study.

### Interventions

All participants were offered behavioural support and nicotine replacement therapy by means of a skin patch, tailored for all participants by phenotype (daily cigarettes smoked). The trial interventions comprised the communication that the prescribed dose for oral NRT treatment was based on either genotype (intervention) or phenotype (comparison).

Support for behavioural change was based on withdrawal orientated therapy [23] and was provided for all participants twice prior to quit day and weekly thereafter until four weeks after quitting and then once more eight weeks after quitting. All nurses were trained to give behavioural support to NHS standards [24]. The support lasted 10–30 minutes, depending upon progress and stage of the quit attempt. It was identical in both arms, except as described below.

Nicotine replacement therapy (NRT) was offered to all participants. Participants, regardless of group allocation, were prescribed a nicotine patch dose based on daily cigarette consumption. Smokers of 15 or more cigarettes daily were prescribed 21 mg patches and those smoking 10–14 cigarettes a day were prescribed 14 mg patches.

Participants were then prescribed a second oral ‘top-up’ NRT with their choice of whether by gum, lozenge, sublingual tables or inhalation spray. The dose was either 6 or 12 mg of absorbed nicotine. A 2 mg gum leads to about 1 mg absorption, and a 10 mg inhalator cartridge typically delivers about 3 mg. Thus the prescribed doses were either 6 or 12 gum, lozenge, or sublingual tablets, or 2 or 4 inhalator cartridges daily. Combination NRT (typically patch plus short-acting form as here), is approximately 35% more effective than patch alone [25].

### Dose Tailoring

**a) Genotype arm.** The dose of oral NRT in this arm was tailored to likelihood of responding to a higher dose as assessed by gene variant. Participants’ blood or saliva samples were tested for the *OPRM1* Asn40Asp (A118G) variant (rs1799971) using standard methods. Those who were homozygous for the Asn40 variant were prescribed a standard oral NRT dose of 6 mg a day. Those who were heterozygous or homozygous for the Asp40 variant of the gene were prescribed a higher dose of oral NRT of 12 mg a day.
**b) Phenotype arm.** The prescribed dose of oral NRT in this arm was determined using the Fagerström Test for Nicotine Dependence score [26] because there is evidence that more dependent smokers benefit from higher doses [25]. Those scoring less than eight were considered to have lower dependence and were recommended to take a dose of oral NRT of 6 mg a day, while those scoring eight or more were considered to have higher nicotine dependency and were recommended to take a dose of 12 mg a day.

**Communication of the basis for oral NRT.** One day prior to quit day, participants were given their NRT patches and oral NRT and told the basis (genotype or phenotype) for the dose of oral NRT by a research nurse who used a standard script that was similar for both arms (see trial protocol [27]). This was reinforced by a personalised booklet documenting the dose of NRT to use daily and giving the reasons for that dose including the physiological mechanisms by which taking their personalised oral dose would increase their chances of stopping smoking. They were also given an appointment card which summarised their NRT doses and the basis for these (including whether their oral dose was based on genotype (blood test) or phenotype (nicotine dependence score), a weekly diary in which to record the NRT consumed, and their first batches of NRT. The nurse reiterated the rationale for the prescribed dose at each weekly clinic.

### Recruitment and Follow-up Procedures

Potentially eligible smokers were identified from practice registers and mailed a letter from their primary care physician expressing concern about their smoking and offering assistance to quit, with an invitation to participate in the trial. Interested participants were screened for eligibility and then attended eight clinic appointments, held weekly, with a research nurse. The quit day occurred two weeks and a day after baseline. On the third session, one day prior to quit day, the nurse revealed the randomisation and told the participant the rationale for their dose. Abstinence was recorded weekly and verified by exhaled carbon monoxide (CO).

#### Adherence checks

The nurse assessed the NRT used from the diary and a count of remaining NRT, and the NRT required for the forthcoming week was dispensed.

#### Fidelity checks

The third clinic visits were audio-recorded to assess fidelity to the communication protocol.

#### Non-attenders

When a participant did not attend a clinic, the nurse made three attempts to contact him or her, after which it was assumed that the participant had ceased trying to quit smoking. Adherence was counted as zero for the remainder of the four weeks unless he or she subsequently returned.

#### Longer term follow-up

All participants were contacted six months following their quit date, either by telephone or by post. Follow-up questionnaires were completed and, in those indicating continued abstinence, a salivary sample was requested by post and subsequently analysed for cotinine.

### Outcomes and Follow-up Primary Outcomes

The primary outcome for hypothesis I was adherence, the natural proximal behaviour for the intervention. While the trial was not powered to detect a difference in long term behavioural outcomes related to smoking, prolonged abstinence was measured at six months post quit date.

#### Adherence to prescription of NRT over 28 days

Adherence to the prescription of NRT was assessed at 28 days following the quit date. It was defined as the proportion of all NRT prescribed (in milligrams) that was consumed on each day, averaged over this time period. Any over-consumption on a day, relative to the amount prescribed, was defined as 100% adherence. Consumption was measured using pill counts and participants’ self-report, recorded in a daily diary. Quality of data was categorised into ‘high’ and ‘lower’ quality. High quality data consisted of i) adherence validated by both the self-report daily diary and the pill count, or ii) data reporting the resumption of smoking, whereby the participant informed the research team that they had abandoned their quit attempt. ‘Lower’ quality data consisted of all other permutations.

#### Motivation to make further quit attempts

This was assessed in those who had returned to smoking at six months follow-up. The measure comprised four items, with response options provided on seven-point rating scales assessing likelihood of a further quit attempt in the following four weeks. Mean values on a composite scale (Cronbach’s alpha  =  0.91) were positively scored ranging from low (1) to high (7) motivation to quit in the next four weeks.

### Secondary Outcomes

#### Adherence to prescription of NRT over 7 days

It was expected that the behavioural effects of the intervention would be stronger in the first week of the quit attempt as fewer participants would have resumed smoking. Adherence to prescription of NRT was therefore assessed at 7 days, using the same approached as described above for 28 days.

#### Abstinence from smoking

This was assessed using the Russell standard procedures [28] counting participants lost to follow up as being smokers and smoking status was verified biochemically. At 28 days abstinence was defined as fewer than five cigarettes in the past two weeks verified by CO<10ppm. At six months, it was prolonged abstinence since the start of week 3, with fewer than five cigarettes smoked and verified by cotinine<15 ng/ml.

### Additional Outcomes

#### Non-use of NRT

We report the proportion at 28 days who had consumed no NRT during the trial.

#### Use of NRT beyond 28 days

At six months, we asked participants whether their use of NRT had continued beyond the 28-day treatment period with responses coded as ‘yes’ or ‘no’.

#### Anxiety

At baseline, one week and six month follow-up, anxiety was measured using the six-item, short-form of the state scale of the Spielberger State-Trait Anxiety Inventory (STAI-6) [29].

### Sample Size

The detectable effect size was chosen to be 7.5% difference in NRT consumption over the 28-day period of NRT prescription, equivalent to a two-day difference. 630 participants provided 90% power using a two-sided unpaired t-test at the 5% level of significance, allowing for 20% dropout. The variability in this skewed outcome and the appropriateness of a t-test were evaluated using a previous simulation of daily prescribed NRT patch use. On this basis we established that the sample size was large enough to meet the assumptions required of a t-test to compare mean adherence between study arms [30]. Clustering was not allowed for in the analysis: the mean cluster size was anticipated to be close to one [31] and was confirmed to be 1.05 participants per family.

### Randomisation

Randomisation was stratified by cigarettes smoked per day (10–14, 15+), FTND score (<8, 8–10), and four nurses. Within each stratum, a randomisation sequence was computer generated using random selected block sizes with a one-to-one allocation ratio. Families were allocated as clusters to the same arm to avoid contamination [31]. The trial statistician generated the sequences and received the stratifier data and participant and family identifier required to randomise participants, and participant date of birth to confirm group allocation at trial closure. Allocations were made in one central location, separate from trial co-ordination, database, and participant enrolment.

### Blinding

The randomisation sequence was revealed sequentially and concealed from the trial team, nurses and participants. However, after assignment to group, nurses, participants, and the trial team were inevitably not blind to allocation. Nurses were informed which dose and communication type they should use before the participants arrived for visit 3 (one day prior to quit date) and the rationale for the prescription was given weekly to participants. Group allocation was concealed from the research team collecting secondary outcome data on self-reported smoking status.

### Statistical Analysis

Intention-to-treat analyses were conducted using SPSS version 15. Adherence outcomes were analysed by comparing adherence to NRT between the two arms by using an independent samples *t*-test and estimating the 95% confidence interval for the between-arm difference in mean NRT consumption. Motivation to make another quit attempt was compared between arms in the subgroup that were not abstinent at six months follow-up, using an independent samples *t*-test.

Smoking abstinence was assessed by comparing the proportion in each arm that were abstinent using a chi-squared test and estimating the difference in proportions with the 95% confidence interval.

Further details on the methods used in this trial are reported elsewhere, in the trial protocol [27].

## Results

### Participant Flow

**Figure 1:**
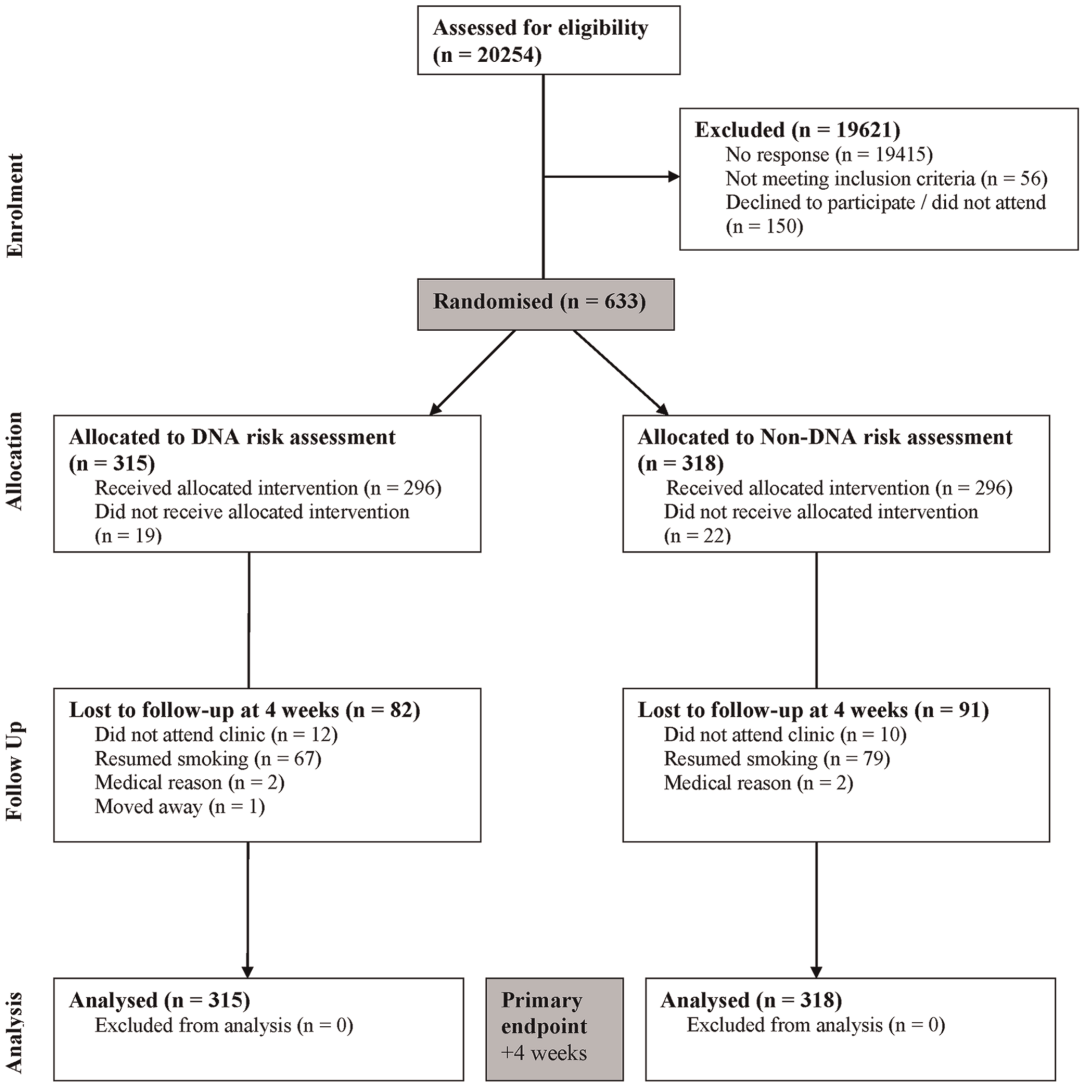
CONSORT flow diagram.

This is shown in Figure 1. Of 20,254 smokers identified, 19,415 did not respond.

Of the 839 who did respond, 206 were deemed ineligible, declined to participate or failed to attend the first clinic appointment required for trial recruitment. This resulted in 633 eligible patients who agreed to participate and who were randomised (3.1%).

### Recruitment

The overall period of recruitment and follow-up ran from June 2007 – September 2009.

### Baseline Data

Baseline demographic characteristics of the two trial groups were similar. Smoking characteristics were similar across groups, including genotype distributions and NRT prescribed (Table 1).

**Table 1:** Demographic characteristics and baseline measures of smoking-related and other variables.

	Genotype (n = 315)	Phenotype (n = 318)
**Demographic characteristics**		
Gender (%(n))		
Male	46.0 (145)	45.3 (144)
Female	54.0 (170)	54.7 (174)
Age (m(sd))	46.9 (13.4)	47.7 (13.2)
Ethnicity (%(n))		
White	89.5 (282)	90.9 (289)
Black	3.5 (11)	1.9 (6)
Asian	2.8 (9)	1.3 (4)
Other	3.5 (11)	4.1 (13)
Missing	0.6 (2)	1.9 (6)
SES (%(n))		
Group 1: most deprived	8.9 (28)	7.9 (25)
Group 2	20.3 (64)	22.6 (72)
Group 3	33.3 (105)	33.3 (106)
Group 4: least deprived	32.7 (103)	32.4 (103)
Missing	4.8 (15)	3.8 (12)
Weight in kg (m(sd))	77.2 (17.7)	77.0 (18.2)
**Baseline smoking variables**		
Fagerström (m(sd))	5.6 (2.1)	5.5 (2.3)
Fagerström (%(n))		
Score <8	81.0 (255)	80.8 (257)
Score 8+	19.0 (60)	19.2 (61)
Number of cigarettes smoked per day (m(sd))	20.5 (8.7)	21.1 (8.5)
Number of cigarettes smoked per day (%(n))		
10–14 cigarettes	19.4 (61)	21.4 (68)
15+ cigarettes	80.6 (254)	78.6 (250)
OPRM1 status (%(n))		
Asn/Asn	81.0 (255)	75.8 (241)
Asn/Asp	18.7 (59)	21.7 (69)
Asp/Asp	0.3 (1)	0.3 (1)
Missing	0	2.2 (7)
Smoking – previous quit attempt (%(n))		
Never tried to quit	28.3 (89)	22.7 (72)
≤1 week or less	13.7 (43)	12.9 (41)
>1 week, ≤1 month	11.4 (36)	13.8 (44)
>1 month, ≤6 months	28.3 (89)	30.2 (96)
>6 months, ≤12 months	9.2 (29)	6.6 (21)
>1 year	8.3 (26)	13.2 (42)
Missing	1.0 (3)	0.6 (2)
Prescription received (%(n))		
Standard patch & standard oral NRT (20 mg)	15.2 (48)	20.1 (64)
Standard patch & higher oral NRT (26 mg)	4.1 (13)	1.3 (4)
Higher patch & standard oral NRT (27 mg)	65.7 (207)	57.2 (182)
Higher patch & higher oral NRT (33 mg)	14.9 (47)	21.4 (68)
Total dose prescribed (mg) (m(sd))	26.8 (3.6)	26.9 (4.2)
**Other baseline variables**		
State anxiety (STAI-6) (m(sd))	38.0 (11.9)	38.5 (13.6)

### Numbers Analysed

We analysed the primary endpoint for all 633 randomised individuals using an intention-to-treat analysis, with missing data regarded as representing zero adherence.

### Outcomes (see Table 2)

**Table 2:** Medication adherence, motivation to quit again and smoking abstinence.

	Genotype n = 315	Phenotype n = 318	Difference (G – P) in mean or proportion (95% CI) and *p*-value
**Primary Outcomes**			
Proportion of all prescribed NRT consumed over 28 days (m(sd))	68.5 (36.3)	63.6 (39.0)	5.0 (−0.9, 10.8) *p* = 0.098
Motivation to make another quit attempt (m(sd))	4.4 (2.0)	4.7 (2.0)	−0.3 (−.7,.2) *p* = 0.228
**Secondary Outcomes**			
Proportion of all prescribed NRT consumed over first 7 days (m(sd))	75.4 (34.1)	69.5 (37.2)	5.8 (0.3, 11.4) *p* = 0.040
28-day prolonged abstinence, validated (%(n))	47.9 (151)	46.2 (147)	1.7, (−6.1, 9.5) *p* = 0.667
6-month prolonged abstinence, validated (%(n))	13.7 (43)	7.9 (25)	5.8 (1.0, 10.7) *p* = 0.018

### Primary Outcomes

#### Adherence to prescription of NRT over 28 days

**Figure 2:**
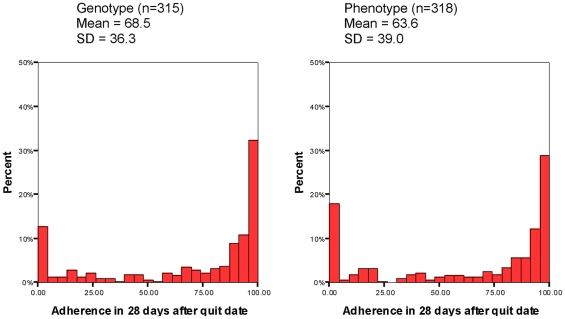
Distribution of adherence to NRT over 28 days after quit date by trial arm.

The proportion of prescribed NRT consumed in the first 28 days following quitting was not significantly different between groups: (68.5% of prescribed NRT consumed in the genotype vs. 63.6%, in the phenotype group, difference  =  5.0%, 95% CI −0.9,10.8, *p  = * 0.098), equivalent to 1.5 days or 1.4 mg per day of extra adherence over the 28-day period. Adherence data were classified as high quality for 68.1% of assessments. *See* *Figure 2*.

#### Motivation to make further quit attempts

For this analysis, the number included was 331 (165 in the genotype arm and 166 in the phenotype arm). Of the 302 participants not included, 167 were excluded as they self-reported abstinence and 135 were lost to follow-up. Participants contacted who were not abstinent at six months (n = 331) showed similar moderate levels of motivation to make another quit attempt in the next month (mean 4.4 vs. 4.7; difference  =  −0.3, 95% CI −0.7,0.2, *p*  =  0.23). Differences between trial arms were small (0.3 points on this composite scale with 1 to 7 range, 2.0 SD (and mid-value 4.0)) and not statistically significant with a narrow confidence interval.

### Secondary Outcomes

#### Adherence to prescription of NRT over 7 days

Analysis of adherence at seven days demonstrated a small effect, similar to that observed over 28 days, which was statistically significant (75.4% consumed vs 69.5%; difference  =  5.8%, 95% CI 0.3,11.4, *p*  =  0.040).

#### Abstinence from smoking

Participants in the genotype arm were significantly more likely to be abstinent at six months (13.7% (43/315) vs. 7.9% (25/318); difference  =  5.8%, 95% CI 1.0,10.7, Relative Risk (RR)  =  1.74 (95% CI 1.09,2.76), *p*  =  0.018) compared with those in the phenotype arm. This analysis was based on biochemically validated non-smokers, comprising 56.7% (68/120) of those participants who self-reported 6-months abstinence: 61.4% (43/70) of those in the genotype arm, and 50.0% (25/50) of those in the phenotype arm. There was no significant difference between arms in validated abstinence at 28 days (47.9% (151/315) vs. 46.2% (147/318); difference  =  1.7%, 95% CI: −6.1,9.5, RR = 1.04 (95% CI 0.88,1.22), *p*  =  0.67).

### Additional Outcomes

Participants in the phenotype arm (16% (52/318)) were more likely than those in the genotype arm (10% (32/315)) to take no NRT: χ^2^(1)  =  5.27, *p*  = .022. This difference remained when those who did not receive the intervention were removed from the analysis (11% (34/318) *vs* 6% (19/315): χ^2^(1)  =  4.48, *p*  = .034).

Participants in the genotype arm were significantly more likely to report continuing use of NRT beyond the 28-day treatment period (74.2% (190/256) vs. 64.1% (152/237); χ^2^(1)  =  5.89, *p*  = .015).

#### Harms

Three serious adverse events were reported, all for participants in the genotype arm (acute respiratory distress syndrome with underlying pneumonia; right sided weakness (possible minor stroke); leg fracture when hit by vehicle). None was plausibly related to the prescribed NRT medication and in all cases patients recovered without sequelae. At baseline, there was no difference between groups in anxiety. Similarly, there were no differences at one week or six month follow-up.

#### Fidelity to protocol

Sessions in which the differential basis for tailoring of oral NRT was communicated were tape-recorded. Assessment of a subsample of randomly selected recordings was conducted (by CH) to assess the fidelity to the clinical protocol of the intervention delivery. This was deemed acceptable in all cases, with delivery of all key components.

## Discussion

### Principal Findings

We found a small, statistically non-significant effect on 28-day adherence of communicating a tailoring of oral dose of NRT to genotype rather than phenotype. This represented 1.5 days, or 1.4 mg per day difference between groups, in mean NRT consumed over a 28 day period. No adverse effects on motivation in those failing to quit were detected. Neither trial hypothesis was supported.

### Interpretation of Study Results

Taken together with the secondary outcomes, our results are consistent with the intervention having a small positive effect upon adherence amongst these participants. The evidence supporting this effect is provided by the 95% confidence interval, with the upper limit of 10.8% being consistent with there being a small positive effect of the intervention on the primary outcome. The confidence interval is also consistent with there being a zero effect of the intervention, but it is inconsistent with anything other than a minute negative effect on smoking cessation. Further evidence to support the intervention having a small effect upon adherence is provided by the secondary outcome of NRT adherence at 7 days, which is statistically significant, and a the biochemically validated prolonged abstinence at 6 months. The higher abstinence observed in the genotype group may in part have been due to the higher proportion of participants in that group who initiated at least some use of NRT as part of their quit attempt (see Figure 2) as well as the higher proportion who reported using NRT beyond the 28 day treatment period. These findings suggest that the intervention may have had an effect both on initiation of the use of NRT and treatment persistence. The factors that might help interpret these observations will be explored in a separate paper.

This first empirical test of the behavioural impact of pharmacogenomics reduces the uncertainty around the possible size of impact. The effect on adherence, if there is one, is likely to be smaller than the two day difference in adherence at one month on which we powered the study.

Overall, 11% of participants (68/633) were abstinent at six months. The difference in abstinence between groups at six months is larger than would be expected from the small observed difference in adherence to NRT at 28 days and the negligible difference in short-term abstinence. If the impact of the intervention on abstinence is not explained by NRT adherence over 28 days, there are a number of other explanations. First, this may be a chance finding. Second, this may be due to bias in the conduct of the study; however randomisation was effective, there was no unequal attrition across groups and any bias might be expected to be apparent during behavioural support and not after it. Third, this may be a valid finding, not mediated by adherence to NRT during the first 28 days of a quit attempt. Whilst we did not assess adherence after 28 days in detail, during the six month telephone follow-up we asked participants whether they had consumed NRT beyond the initial treatment period, with more in the genotype arm reporting such use than those in the phenotype arm. This finding should, however, be considered with caution. The measure used relies on retrospective long-term recall and the analysis was not specified prior to conducting the study. The observed effect on long-term abstinence may also reflect impact of the intervention on other smoking-related behaviours which we did not assess. Further analyses of these data, modelling causal effects of psychological and other variables collected during the trial, may shed some light on this.

We found no effect on continuing intention to quit smoking from informing participants that their oral NRT dose was tailored to genotype rather than phenotype. In particular people in the genotype arm who were not abstinent at six months were as likely to plan a further quit attempt as those in the phenotype arm. Despite the plausibility and frequently expressed concerns for such an effect, these findings are consistent with other, related evidence synthesised in a systematic review which provide no evidence to suggest that communicating personalised genetic risk information engenders feelings of fatalism [22]. The results of the current study add to this by showing, we believe for the first time, that feelings of fatalism are also not engendered by failure to change behaviour following personalised genetic risk information.

### Strengths and Limitations of the Study

The study has several strengths. First, it is novel, being the first to test the behavioural impact of pharmacogenomic tailoring of medication. Second, we used a robust design with evidence of success in balancing the two groups for key confounders including baseline smoking variables and genotype. Third, we powered for a plausible and clinically important difference. Fourth, we used objective measurement with biochemical verification of smoking status, which strengthens the interpretation of smoking abstinence.

There are also limitations. The primary endpoint was not smoking abstinence which would have required a larger trial. Adherence was chosen as the endpoint as the impact on smoking cessation of the intervention was predicted to work through adherence to NRT. The measure of adherence was based in part on self-report of medication use. Consumption was assessed using ‘pill’ counts as well as diary records and the practice nurse checked for discrepancies at each clinic visit and reconciled them with participants. We used more than one index of adherence to increase reliability. While the measures used lack the precision of some electronic devices, such devices cannot be used to measure length of exposure to patches or gums. Regarding the second hypothesis, we did not measure quitting behaviour beyond six months to assess whether motivation to make a quit attempt in those who were not abstinent reflected in actual behaviour. Motivation is, however, a reasonable indicator of subsequent behaviour [32].

The experimental design constrains the conclusions that can be reached. The design that was ultimately chosen does not clearly separate the relative influences of communication of the basis for prescribing and dose tailoring. The most attractive experimental design to investigate the effects of communication would have required us to use the same dosing algorithm in both arms but to randomise each arm to receive different information on the method used to tailor the dose. Clearly this would have necessitated deceiving participants and we regarded this as unacceptable. We considered seeking informed consent to this approach, but rejected it because of concerns that this might undermine trust in the trial as a whole. The design is limited in two further ways that may have produced a conservative estimate of the intervention effect. First, all participants received a prescription tailored to phenotype for transdermal NRT which may have served to dilute the impact of the intervention. The design was further limited by not including a group that received standard care only, or a group that received no behavioural treatment *i.e.* only tailored NRT prescriptions. Future studies might usefully assess the impact of communicating treatments tailored by genotype in different treatment contexts.

### Generalisability

As expected, a minority of smokers (3.1%) who received an invitation to join a study of smoking cessation did so. In the UK around 5% of smokers a year use behavioural support and medication provided by NHS clinics. That our uptake is similar suggests that the results are likely to be generalisable to smokers trying to stop smoking with support and medication. It was our impression that people participated primarily to stop smoking rather than to participate in research.

### Implications for Clinicians and Policy Makers

The results of the current study suggest that communicating to smokers that their NRT dose has been tailored by genotype is unlikely to cause harm. The effects on adherence were small at best, and the effects on abstinence and their mechanism, unclear. If the observed effects on smoking cessation at six months are replicated, however, genotype tailoring could contribute positively at a population level to smoking cessation interventions. Any possible contribution of genotype to tailored prescribing in smoking cessation should not detract from the increasing evidence for prescribing larger doses of NRT for more dependent smokers [25]. Beyond smoking cessation, the current study is broadly consistent with effects of DNA-based risk communication observed across a range of health behaviours [33], suggesting that these effects are likely to be small or non-existent, and not have the aggregate behavioural impact that many anticipated.

### Unanswered Questions and Future Research

Unanswered questions arising from these findings include: are the effects on smoking abstinence at six months real and how are they mediated, and, to what extent does the behavioural response to the communication of tailoring of medication to genotype vary by disease or service context?

### Conclusion

This first empirical test of the behavioural impact of pharmacogenomics suggests that the impact on adherence to NRT may be small, at best. Further studies are warranted given the design constraints and the potential for behavioural impact with potential clinical significance. No adverse effect was detected on motivation to start another quit attempt amongst those who were not abstinent at six months. The unexpected higher rate of abstinence at six months in the genotype arm needs further investigation.

## Supporting Information

Checklist S1 **CONSORT Checklist.**
(DOCX)

Protocol S1 **Trial Protocol.**
(PDF)
