# Supplementary material for: Effect on Adherence to Nicotine Replacement Therapy of Informing Smokers Their Dose Is Determined by Their Genotype: A Randomised Controlled Trial
Source: PLoS One. 2012 Apr 11;7(4):e35249. doi: 10.1371/journal.pone.0035249 (PMC3324463; doi:10.1371/journal.pone.0035249)
Supplement: Protocol S1 — Trial Protocol. (PDF) [file pone.0035249.s002.pdf]

Marteau et al. BMC Public Health 2010, 10:680
http://www.biomedcentral.com/1471-2458/10/680

BMC Public Health

STUDY PROTOCOL

Open Access

# Trial Protocol: Using genotype to tailor prescribing of nicotine replacement therapy: a randomised controlled trial assessing impact of communication upon adherence

Theresa M Marteau[1*], Marcus R Munafò[2], Paul Aveyard[3], Chloe Hill[1], Sophia Whitwell[1], Thomas A Willis[1], Rachel A Crockett[1], Gareth J Hollands[1], Elaine C Johnstone[4], Alison J Wright[5], A Toby Prevost[5], David Armstrong[5], Stephen Sutton[6], Ann Louise Kinmonth[6]

## Abstract

**Background:** The behavioural impact of pharmacogenomics is untested; informing smokers of genetic test results for responsiveness to smoking cessation medication may increase adherence to this medication. The objective of this trial is to estimate the impact upon adherence to nicotine replacement therapy (NRT) of informing smokers that their oral dose of NRT has been tailored to a DNA analysis. Hypotheses to be tested are as follows:
I Adherence to NRT is greater among smokers informed that their oral dose of NRT is tailored to an analysis of DNA (genotype), compared to one tailored to nicotine dependence questionnaire score (phenotype).
II Amongst smokers who fail to quit at six months, motivation to make another quit attempt is lower when informed that their oral dose of NRT was tailored to genotype rather than phenotype.

**Methods/Design:** An open label, parallel groups randomised trial in which 630 adult smokers (smoking 10 or more cigarettes daily) using National Health Service (NHS) stop smoking services in primary care are randomly allocated to one of two groups:
i. NRT oral dose tailored by DNA analysis (*OPRM1* gene) (genotype), or
ii. NRT oral dose tailored by nicotine dependence questionnaire score (phenotype)
The primary outcome is proportion of prescribed NRT consumed in the first 28 days following an initial quit attempt, with the secondary outcome being motivation to make another quit attempt, amongst smokers not abstinent at six months. Other outcomes include adherence to NRT in the first seven days and biochemically validated smoking abstinence at six months. The primary outcome will be collected on 630 smokers allowing sufficient power to detect a 7.5% difference in mean proportion of NRT consumed using a two-tailed test at the 5% level of significance between groups. The proportion of all NRT consumed in the first four weeks of quitting will be compared between arms using an independent samples *t*-test and by estimating the 95% confidence interval for observed between-arm difference in mean NRT consumption (Hypothesis I). Motivation to make another quit attempt will be compared between arms in those failing to quit by six months (Hypothesis II).

**Discussion:** This is the first clinical trial evaluating the behavioural impact on adherence of prescribing medication using genetic rather than phenotypic information. Specific issues regarding the choice of design for trials of interventions of this kind are discussed.

**Trial details:** Funder: Medical Research Council (MRC)

* Correspondence: theresa.marteau@kcl.ac.uk
[1]King's College London, Psychology Department (at Guy's), Health Psychology Section, 5th Floor Bermondsey Wing, Guy's Campus, London SE1 9RT, UK
Full list of author information is available at the end of the article

BioMed Central

© 2010 Marteau et al; licensee BioMed Central Ltd. This is an Open Access article distributed under the terms of the Creative Commons Attribution License (http://creativecommons.org/licenses/by/2.0), which permits unrestricted use, distribution, and reproduction in any medium, provided the original work is properly cited.

Grant number: G0500274
ISRCTN: 14352545
Date trial stated: June 2007
Expected end date: December 2009
Expected reporting date: December 2010

## Background

Adherence to nicotine replacement in smoking cessation is widely perceived to be problematic; educating smokers about nicotine replacement use with generic or tailored feedback does not readily alter behaviour [1,2]. There are, however, high expectations of the potential for DNA-based risk information to motivate behaviour change more strongly than other types of risk information [3-5]. Such expectations are consistent with theories of attitude change which predict that the greater the personal salience of information, such as information regarding one's own DNA, the greater its impact [6]. We present here a protocol for a randomised controlled trial assessing the impact on adherence of prescribing a tailored oral dose of nicotine replacement therapy (NRT) based on genotype assessing smokers' receptivity to nicotine, compared with a prescription tailored on nicotine dependence (phenotype). The behavioural effect will be measured by the proportion of all prescribed NRT consumed.

It is well-established that both the initiation and maintenance of smoking have high heritability [7-9]. There is also growing evidence that some of the variability in responsiveness to smoking cessation pharmacotherapy, such as NRT or bupropion, is explained by genotype [10-12]. The Asn40Asp (A118G) polymorphism (rs1799971), found in exon I of the *OPRM1* gene, has been associated with functional changes in mu-opioid receptors [13]. This mis-sense SNP leads to the substitution of an asparagine (Asn) for an aspartate (Asp) at position 40 in the amino acid sequence. The Asp (G) allele has been shown to bind beta-endorphin three times more strongly than the Asn (A) allele [14], although two replication studies failed to find this [15,16]. The Asp (G) allele has also been associated with reduced expression of the receptor [16]. Thus, for example, smokers with a particular variant in the mu-opioid receptor (*OPRM1*) gene (Asp40, present in about 25% of the population) that regulates the binding affinity of beta-endorphins, have been reported to show double the short term quit-rates when using a form of NRT with higher levels of replacement compared with NRT that results in lower levels of replacement [17]. Those homozygous for the more common variant (Asn40) were equally likely to stop smoking regardless of the level of NRT replacement. These results support the hypothesis that smokers with one or more copies of the Asp40 variant are more likely to stop smoking following higher doses of NRT than are smokers without this variant. Although this association was not replicated in a more recent study [18], the earlier research provides face validity for an exploration of the motivating impact of such DNA based information on smoking cessation.

Genetic tests are now available via the Internet (e.g. Respiragene http://www.synergenz.com), which, it is claimed, may be used to identify the optimal pharmacotherapy for an individual wishing to stop smoking or motivate an individual to stop smoking. The impact of such testing on smoking cessation behaviour is currently unevaluated but it has the potential to increase quit rates in two key ways. Firstly, by more effective prescribing i.e. prescribing that is tailored to the individual's nicotine metabolism to avoid over-prescribing with consequent lack of adherence or under-prescribing with consequent urges to smoke; and secondly, by increasing expectations of treatment effectiveness, and hence motivation to comply with the treatment. The focus of the current trial is upon the latter. There is growing evidence to suggest that perceiving a health problem to have a genetic cause increases the perceived effectiveness of taking medication to deal with the problem [19]. This has been documented for depression, heart disease and stopping smoking [20-23]. Given perceived treatment effectiveness predicts treatment use, tailoring treatment on the basis of genetic testing has the potential to improve treatment outcomes by increasing adherence. These observations inform the scientific rationale and principal hypothesis for the current trial.

A preliminary study suggests that prescribing based on genetic testing has the potential both to facilitate cessation by increasing the attractiveness of effective pharmacological treatments, as well as to undermine it, by decreasing the perceived importance of willpower in smoking cessation [23]. In this experimental analogue study, smokers who were asked to imagine that they had a gene variant that predicted a good response to bupropion, a medicine used to facilitate smoking cessation, were more likely to select the use of bupropion, the more effective treatment given their gene variant, to assist them in quitting. They were, however, less likely to perceive "willpower" as being important [23]. Other studies also report that medication is perceived as more

effective when a condition is perceived as genetic [20-22]. In one such study, smokers who were randomised to receive feedback of genetic susceptibility to lung cancer perceived NRT as more helpful when quitting, compared to the group not receiving genetic feedback [24].

We predict that prescribing tailored to genotype increases adherence to NRT compared with prescribing tailored to phenotype, by strengthening the perceived effectiveness of the medication, a good predictor of adherence [25]. Consumption of NRT directly increases the likelihood of sustained smoking cessation; this being dose-dependent in that the more NRT consumed, the greater the chance of sustained cessation [26,27]. It is the most popular medication used in UK NHS clinics [28].

Furthermore, given the common representation of genes as deterministic, conferring immutable effects, it is important to document the impact of DNA testing for nicotine responsiveness in the longer term upon attributions for failure to quit smoking among those smokers who do not quit. Informing patients that their prescription is tailored to their genotype may have negative effects through engendering a sense of fatalism, which is associated with perceiving a genetic cause to a health problem [29]. Such fatalistic responses are predicted to result in reduced motivation in this group to make a future quit attempt (amongst those who fail to quit by six months).

### Objective and hypotheses

We will estimate the impact upon adherence to NRT of informing smokers that their oral dose of NRT has been tailored on the basis of DNA analysis.

The trial tests two hypotheses:

I Adherence to NRT is greater among smokers who are informed their oral dose of NRT is tailored to an analysis of DNA (genotype), compared to one tailored to nicotine dependence (phenotype).

II Amongst smokers who fail to quit at six months, motivation to make another quit attempt is lower when informed that their oral dose of NRT was tailored to genotype rather than phenotype.

While the trial is not powered to assess long term behavioural outcomes related to smoking, prolonged abstinence is measured at six months post quit date.

## Methods/Design

### Trial Design (*see Figure* 1)

An open label, parallel group, randomised controlled trial in which participants were randomly allocated on a 1:1 basis to one of two groups:

i. NRT oral dose tailored by DNA analysis, or

ii. NRT oral dose tailored by nicotine dependence score

### Study Setting

The trial is taking place in National Health Service (NHS) smoking cessation clinics in primary care. These provide a combination of weekly behavioural support and pharmacotherapy to assist smokers to quit. Participants are being recruited from 29 primary care practices in two English cities, Birmingham and Bristol.

### Participants

The sample will comprise 630 smokers using NHS stop smoking services within primary care. To be included, participants must be aged 18 years or older and a regular cigarette smoker who wants to stop smoking. A 'regular smoker' is defined here as someone who has smoked an average of 10 or more cigarettes per day (including roll-ups) over the preceding 12 months. They must also be able to give informed consent to participate as well as to be able to complete the study questionnaires, either alone or with assistance.

Exclusion criteria:

1. Cigar, pipe and oral tobacco users who do not also smoke 10 or more cigarettes per day.
2. Those who meet the criteria contra-indicating NRT use, as described in the Summary Product Characteristics, updated by recent MHRA guidance. Although not all forms of NRT are contra-indicated in pregnancy or lactation, the metabolism of nicotine changes and there remain concerns about safety in this group which include the avoidance of 24 hour patches as described in our protocol. Therefore pregnant or lactating women or those who plan to become pregnant during the course of treatment will be excluded.
3. Those with previous severe adverse reactions to NRT patch or to oral NRT.
4. Those currently taking either medication for smoking cessation that they are unwilling to stop taking medication with a known influence on smoking cessation that they should not stop (*e.g.* nortriptyline for depression).
5. Those who are non-English speakers.
6. Those deemed unsuitable for the study by their primary care physicians.

### Genetic testing

All participants will give a sample of either blood or saliva for genotyping, although the results are only used for tailoring dose of oral NRT and communicated to participants in the intervention group.

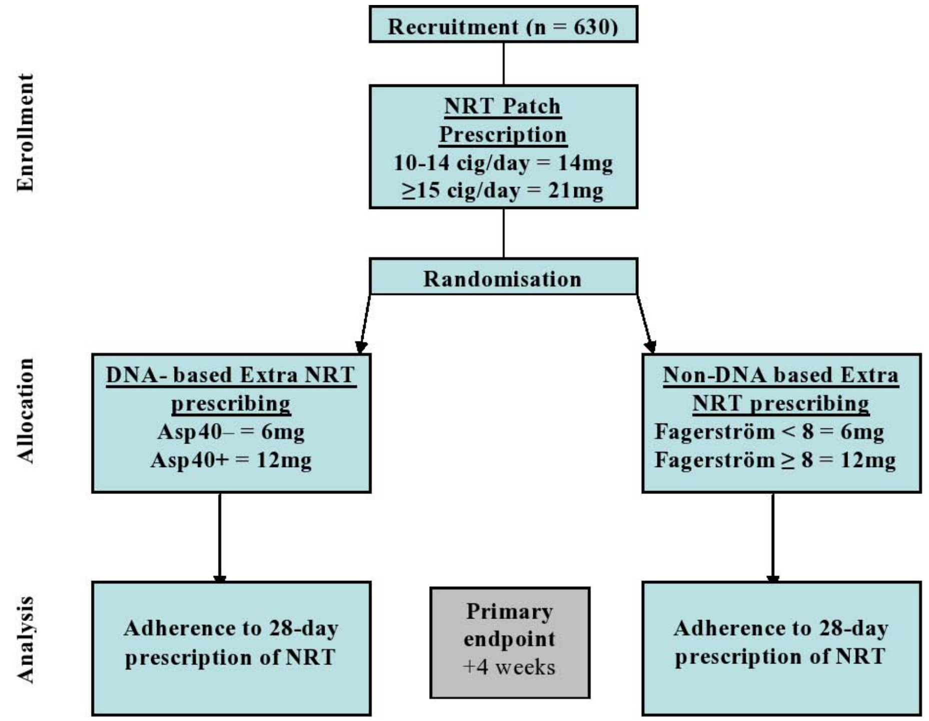


**Figure 1** Trial flow diagram.

This trial tests for variants in the mu-opioid receptor gene, *OPRM1*. It is expected that about 75% will be homozygous for Asn40, with about 25% heterozygous or homozygous for Asp40 (i.e. need the higher dose of NRT) [18]. DNA will be extracted from blood using commercially available methods. From participants who do not want to have a blood test, DNA will be extracted from saliva. Briefly, DNA is extracted using a standard "salting out" technique [30]. For the genotyping of the *OPRM1* A118G polymorphism, PCR is carried out using an allele specific, two-tube primer method. The reaction mixture contained 0.5 μM of each *OPRM1* and control primer, approximately 150 ng of DNA, 200 μM dNTPs, 2 mM $MgCl_2$ and 0.5 units of Taq polymerase in a final volume of 25 μl. After an initial denaturation step for 10 min at 95°C, thermocycling consisting of 30 cycles of 95° C for 20 sec, 68°C for 45 sec, 72°C for 30 sec. A final extension phase of 72°C is followed by cooling of samples to 4°C. PCR products are separated on a 1% agarose gel at 200 V for 30 min and visualised using ethidium bromide. In order for samples to be valid, a 750 bp control product has to be formed in both allele specific reactions. A homozygote is indicated by the presence of a 561 bp product in one of the reactions and a heterozygote is represented by a 561 bp product in both of the reactions.

A greater than 95% success rate is expected for the extraction and amplification of DNA. In the event that DNA is not collected or laboratory procedures fail to extract and amplify the DNA from the sample, the participant will be asked to provide a further sample.

### Trial medication

The trial will take place in the context of NHS stop smoking services, which provide behavioural support and medication to assist smoking cessation. At the time that this trial started, more than 70% of smokers in these clinics used nicotine replacement therapy (NRT) [28]. Current best practice is to use NRT in combination with behavioural support [31,32]. Smokers in the current trial will all be prescribed NRT patches plus oral NRT 'top-up' medication, which is approximately 35% more effective than NRT patch alone [33]. NRT patch dose will be tailored in the same way in both arms of the trial, namely according to how many cigarettes are smoked per day. Oral NRT dose will be determined on a different basis in each arm of the trial: those in the genotype arm will have the dose tailored according to the *OPRM1* variant that they carry; those in the phenotype arm will have the dose tailored according to their nicotine dependence. Details of the basis for these prescriptions are presented below. The aim of treatment is to continue with full dose NRT for the first four weeks after the quit day, after which medication is reduced, according to patient need.

### Interventions

All participants are to be offered behavioural support and nicotine replacement therapy. The intervention to which participants are randomised comprises the communication that their dose for oral NRT treatment is based on either genotype or phenotype.

#### *Support for behavioural change*

This is based on withdrawal orientated therapy [34] and is provided for all participants twice prior to quit day and weekly thereafter until four weeks after quitting and then once more eight weeks after quitting. All nurses have been trained to give behavioural support to NHS standards [35]. The support lasts 10-30 minutes, depending upon progress and stage of the quit attempt.

#### *NRT patch prescription*

All participants smoking 15 or more cigarettes per day are prescribed 21 mg patches; those smoking 10-14 cigarettes per day will be prescribed 14 mg patches.

#### *Oral NRT prescription*

All participants will be offered additional oral nicotine replacement (gum, microtabs, lozenges, or inhalator) with the choice of delivery system left to personal preference. The dose of oral NRT prescribed is based on delivered dose. Thus, a 2 mg gum delivers about 1 mg of NRT into the circulation, and a 10 mg inhalator cartridge delivers about 3 mg of NRT.

***i. Genotype arm (Intervention)*** Participants will be informed that their oral dose of NRT is based on their genetic test result, using the following script:

*"Your extra NRT is based on the results of a genetic test. We did a genetic test on the blood sample that you gave last week. People have different versions of the 'OPRM1' gene. This gene influences how dependent you are on nicotine. There is more information on genes in the leaflet. Based on the results of your genetic test, you are more likely to be successful in stopping smoking if you have a (**standard dose/higher dose**) of extra NRT. Recent research shows that people with your genetic test result are more successful at stopping smoking if they take all of the extra NRT, as well as using their patch.*

*You should wear a new patch for 24 hours each day for at least four weeks. The patch works by releasing a steady dose of nicotine into your blood stream. You have also been given a **(dose/high dose)** of extra NRT. Please use this as well as wearing the patch. You may wish to take it when you get a craving, but you can also take it at other times of the day. Even if you feel you don't need the extra NRT, you should take it. Many quit attempts fail because people don't take enough NRT or stop taking it before they have beaten their withdrawal symptoms. Remember that this dose has been calculated to suit your individual needs - try to stick to this amount each day in addition to wearing the patch."*

Participants with the Asn variant will be advised to take additional NRT equivalent to the delivery of 6 mg of NRT a day. Those with the Asp variant will be advised to take additional NRT equivalent to delivery of 12 mg a day. A rationale booklet will be given to each participant describing:

i. their personalised additional oral dose of NRT based on their genetic test result. Participants with the Asn variant will be informed that they need a standard dose of additional NRT and participants with the Asp variant will be informed that they need a higher dose of additional NRT;
ii. the basis upon which this dose was calculated (*ie* a genetic test to establish which version of the "*OPRM1*" gene they carry);
iii. information about genes;
iv. information about the amount of additional NRT they should take in addition to wearing a new patch every day for at least 4 weeks;
v. the physiological mechanisms by which wearing their NRT patch and taking all their personalised dose of additional NRT each day for four weeks increases their chances of stopping smoking.

***ii. Phenotype arm (Comparison)*** Participants will be informed that their oral dose of NRT is based on their nicotine dependence scores from questionnaire answers, using the following script:

*"Your extra NRT is based on the results of the questionnaire you completed last week. The questionnaire shows how dependent you are on nicotine. Based on the results of this questionnaire, you are more likely to be successful in stopping smoking if you have a (**standard dose/higher dose)** of extra NRT. Recent research shows that people who got this score on the questionnaire are more successful at stopping smoking if they take all of the extra NRT, as well as using their patch.*

*You should wear a new patch for 24 hours each day for at least four weeks. The patch works by releasing a steady dose of nicotine into your blood stream. You have also been given a (**dose/high dose**) of extra NRT. Please use this as well as wearing the patch. You may wish to take it when you get a craving, but you can also take it at other times of the day. Even if you feel you don't need the extra NRT, you should take it. Many quit attempts fail because people don't take enough NRT or stop taking it before they have beaten their withdrawal symptoms. Remember that this dose has been calculated to suit your individual needs - try to stick to this amount each day in addition to wearing the patch."*

Participants scoring less than 7 on the Fagerström Test for Nicotine Dependence (FTND - [36]) are categorised as low dependence and those scoring 8 or above as high

dependence. It is estimated that approximately 25% of the sample will score 7 or higher on the FTND [37]. Drawing on usual practice in which dose is proportional to smoking burden [38], those in the low dependence group will be advised to take an additional amount of top up to deliver 6 mg of NRT. Those in the high dependence group will be advised to take an additional amount equivalent to 12 mg of NRT delivered a day.

Planned interim analysis (overseen by the Trial Steering Committee) including the first 160 participants revealed a substantial difference between the number of participants that obtained the higher dose of additional NRT in the phenotype arm (n = 32) compared to the genotype arm (n = 14). In order to balance the higher and lower dose oral NRT in the two arms, a cut-off of 8 rather than 7 on the FTND will be used for the remainder of recruitment.

A rationale booklet will be given to each participant, describing:

i. their personalised additional oral dose of NRT based on their questionnaire responses. Participants who score less than 8 (or 7, prior to an interim analysis) on the FTND are informed that they need a standard dose of additional NRT; participants who score above the cut-off are informed that they need a higher dose of additional NRT;
ii. the basis upon which this dose was calculated (*i.e.* responses to a questionnaire, revealing how dependent they are on nicotine);
iii. information about the amount of personalised additional NRT they should take in addition to wearing a new patch every day for at least 4 weeks;
iv. the physiological mechanisms by which wearing their NRT patch and taking all their personalised dose of additional NRT each day for four weeks increases their chances of stopping smoking.

## Procedure

### *Participant recruitment*

Smokers listed on the databases of participating general practices will be written to inviting those wanting to stop smoking to come into the trial. Those interested will be invited to contact a research nurse to arrange to attend a clinic at their general practice run by the research nurse.

### *Consent process*

Those wishing to participate will be asked to sign the study consent form. Blood will be taken for DNA testing from both groups and the baseline questionnaire will be completed. A time will be arranged for the nurse to telephone to confirm participation and attendance at the next clinic visit. Those confirming participation will then be randomized.

### *Data collection*

Participants will be asked to attend seven weekly clinic sessions. In the first clinic participants will complete a baseline questionnaire. NRT will be prescribed according to the protocol at the second clinic visit and a quit date agreed. All participants will be given a small card summarising their top up dose of NRT and describing the rationale for this, which will vary according to whether it is tailored by DNA analysis or nicotine dependence. In the third clinic a second questionnaire will be completed. Participants are requested to continue to take their NRT as prescribed for four weeks post quit attempt and to attend weekly clinic visits. All participants will be contacted six months after their quit dates to assess the study endpoints by questionnaires sent by post or administered by telephone.

## Outcomes

### *Hypothesis I*

***i) Primary outcome*** The primary outcome is adherence to a 28-day prescription expressed as the proportion of all NRT prescribed (in milligrams) that is consumed each day in the first four weeks of quitting, averaged over the four week quit period.

The proportion consumed will be measured by self-report daily diary, backed up by pill counts. If pill counts are discrepant with the diary, this will be discussed and reconciled by the research nurse at the weekly clinic visit. The adherence proportion on any day is the ratio of the amount of NRT consumed to the amount prescribed. The denominator of the amount prescribed derives from both NRT patch and top up. For example, for someone prescribed 1 × 21 mg patch and 6 × 1 mg oral NRT doses (equivalent to 6 × 2 mg gums, for example), the denominator is 27 mg. Consumption of the prescribed top ups and any additional top ups taken will count towards the total NRT consumed on a day. Wearing the patch for 24 hours a day will count as the maximum amount of NRT mg prescribed as having been consumed from the patch. If the patch is worn for a fraction of the 24 hours (such as 12 hours) then this fraction of the total NRT mg from the patch will be counted as having been consumed (such as 0.5 × 21 mg for a 21 mg patch worn for 12 hours), pro-rata. If the patch is not worn for 24 hours a day but additional oral NRT is taken then this will count towards the total NRT consumed on that day. For example, if someone prescribed a 21 mg patch and 6 mg of oral NRT, wears their patch for 16 hours a day and uses an additional 7 pieces of gum to make up for the 7 mg of NRT lost by the patch coming off early, this will be treated as 100% adherence (i.e. they have consumed 14 + 6 +7 = 27 mg). NRT consumed above that prescribed on any day could occur through excess top up consumption and will be treated as a daily proportion of 100%. The

daily proportion will be treated as 0% when no NRT is consumed, if, for example, a person has resumed smoking.

Data quality for the primary outcome will be categorised into two main categories as 'high' or 'lower' for analysis. High quality data consists of i) adherence data that are validated by both the self-report daily diary and the pill count at the clinic visit, or ii) data reporting the resumption of smoking, whereby the participant informs the research team that they abandoned their quit attempt and resumed smoking. 'Lower' quality data consists of all other permutations.

It is expected that the behavioural effects of the intervention may be stronger in the first week of the quit attempt as fewer participants will have resumed smoking. Therefore a further short-term outcome is adherence to NRT during the first 7 days of the quit attempt.

***ii) Secondary outcomes***

***Adherence to all NRT prescribed, consumed in the first seven days*** This will be assessed in the same way as described for the primary outcome described above, but using the time period of the first seven days of the quit attempt.

***Validated abstinence from smoking at 28 days and six months*** At six months, 7-day point prevalence and six month prolonged abstinence will be recorded. Point prevalent abstinence is defined as smoking not more than 5 cigarettes for the seven days prior to the assessment confirmed by salivary cotinine < 15 ng/ml. Prolonged abstinence (defined as sustained abstinence after an initial two week grace period as recommended by Hughes and colleagues [39] will be operationalised by: a) self-reported consumption of not more than five cigarettes since the quit date; and b) salivary cotinine levels of less that 15 ng/ml [40]. A 28-day abstinence measure will also be reported, with validation by carbon monoxide less than 10 ppm, with backward inference of missing data from the 6 month measure following Russell standard criteria [40].

#### *Hypothesis II*

***i) Within-subgroup outcome: Motivation to make another quit attempt*** The sub-group comprises those participants who fail to achieve prolonged abstinence at the six month follow-up. The primary outcome within this subgroup is motivation to stop smoking in the next four weeks.

This is measured using the mean score of the following four items measured using 7-point bipolar scales: "Do you intend to stop smoking in the next 4 weeks?"[response scale labelled at each end: "definitely do not" (1) and "definitely do" (7)]: "How likely is it that you will stop smoking in the next 4 weeks?" ["very unlikely" (1), and "very likely" (7)]; "How determined are you to stop smoking in the next 4 weeks?" ["not at all" (1), and "extremely" (7)]; and "How much do you want to stop smoking in the next 4 weeks?" ["not at all" (1), to "very much" (7)].

### Sample size calculation

With 315 participants per arm, there is 90% power to detect a 7.5% difference in the mean proportion of NRT consumed using a two-tailed test at the 5% level of significance. This is equivalent to detecting approximately a two days' worth difference in mean NRT consumption over a 28-day period. The calculation is based on the skewed distribution of days of prescribed NRT patch use reported by Alterman and colleagues [41] where 55% of participants reported high use and the remainder had a uniformly spread distribution of use. Assuming the same mixed distribution, with 50% and 65% of high-use participants and the remainder uniformly distributed in the two arms, the implied difference in mean consumption was 7.5%. By repeated random sampling, the distribution of the mean within each group was close to normal. The estimated pooled within-group standard error was 1.85%, and both Mann-Whitney and unpaired t-tests provided at least 90% power.

### Randomisation

#### *Procedure*

After receiving signed informed consent from a participant at the first clinic appointment (two week pre-quit), the study nurse will telephone the participant to ensure participation and commitment to the quit attempt. If confirmed, the trial co-ordination team (KCL) will send the participant's data that are required for randomisation to the statistical team (Cambridge), and receive back the identity of the allocated group.

#### *Method*

Each participant will be randomised to one of the two arms on a 1:1 basis. The randomisation will be stratified by nurse, cigarettes smoked per day (i.e., 10-14 or 15+) and the Fagerström Test for Nicotine Dependence score (< 7 (< 8) or 7-10 (8-10)). Within each stratum, a randomisation sequence will be generated using blocking, with successive randomly selected blocks sizes of six, eight and ten, in order to minimise predictability of assignment. Families will be allocated as clusters to the same arm to avoid contamination and reflect practice. First participants from families will be assigned a group according to the next blocked allocation in their stratum.

#### *Concealment*

At the start of the trial the randomisation sequence in each stratum will be generated using randomly permuted blocks by the trial statistician and concealed from the trial coordination team, therapists and participants. The statistical team will be given a participant's study identification code and the stratifier data necessary for the participant's randomisation. The only other information sent will be the participant's date of birth

which will allow, at study closure, the agreement between the sequence generated and that used in the trial to be confirmed to have been securely operated.

After assignment, participants' study arm will not be concealed from participant, nurse or researchers. This will include the nurses collecting the primary endpoint data as they are also delivering the intervention. However, group allocation will be concealed from the research team collecting secondary outcome data on self-reported smoking status.

### Fidelity checks

All sessions in which the rationale for the prescription will be presented (*ie* the second session, one-week pre-quit) will be tape-recorded. During the recruitment phase of the trial, assessments of a subsample of randomly selected recordings will be conducted to assess the fidelity of intervention delivery. Subsequently, feedback will be provided to the nurses to optimise intervention delivery. In addition, a sub-sample of these recordings will be randomly selected and transcribed to assess fidelity to the clinical protocol. The tapes will be stored in a locked cabinet until the end of the trial and completion of this analysis. They will be accessible only to the study team.

### Adverse events monitoring

At each of the weekly clinic visits following the quit attempt, participants will complete a schedule of nicotine overdose symptoms. This assessment is recorded in the Case Report Form, together with details of any action taken (e.g. continuing with prescribed dose/directing the participant to a lower dose). In addition, at each weekly clinic visit, participants will be asked about the occurrence of any significant side effects from their treatment. The trial nurses will enquire about any adverse events to determine their severity and provide appropriate advice about their management (e.g. rotating the NRT patch site or use of emollients for skin reactions). Examples of adverse events include symptoms of nicotine overdose (e.g. vomiting, headaches) and adverse reactions to NRT (e.g. skin and subcutaneous tissue disorders). All of these events as well as all comments made by participants and health care professionals will be logged in the Trial Log File. Each event will be given an individual number and logged in a computer database. Any follow-up or further action needed will also be logged. No personal information will be included unless absolutely necessary. The Trial Log File will be stored in a locked office.

Any incident judged to be serious by the principal investigator and the research team will be reported to the trials independent Data Monitoring Committee within 24 hours. Any serious adverse events will be reported to Medicines and Healthcare Products Regulatory Authority (MHRA).

### Research Governance

This study is classified as a Clinical Trial of an Investigational Medicinal Product (CTIMP) as it investigates the efficacy of a medicine for which the prescribed dose is determined by the results of genetic testing. Although the trial participants may be prescribed NRT without participating in the trial, the dosage and timing of the prescription is determined by a clinical protocol and therefore the Medicines for Human Use (Clinical Trials) Regulations (2004), apply. Further information is available from the MHRA algorithm at http://www.ct-toolkit.ac.uk. MHRA approval has been granted (MHRA ref: 24570/0002/001-0001; Eudract no: 2006-000106-24).

Ethical approval for the trial has been granted (Hertfordshire 1 Research Ethics Committee, ref: 06/Q0201/21, approved $26^{th}$ June, 2006). R&D approval has been granted from the relevant PCTs in Birmingham and Bristol where the interventions will take place.

### Statistical Analysis

All analyses will be primarily on an intention-to treat-basis, with the intention to treat population defined as those randomised into the trial. This compares the pragmatic policies represented by the two intervention arms amongst all those eligible and consenting. This will be supported by an explanatory per protocol analysis, with the per protocol population defined as all participants with the exclusion of those who chose not to attend to receive their allocated intervention.

#### *Hypothesis I: Analysis of primary outcome*

For the analysis of the primary outcome, the proportion of all NRT consumed in the first 28 days of quitting will be compared between arms using an independent samples t-test and by estimating the 95% confidence interval for the observed between-arm difference in mean of proportion of NRT consumed (Hypothesis I). This will be confirmed using the nonparametric Bootstrap BCA method for estimating the difference between two means with confidence interval.

#### *Secondary analyses*

The proportion of all NRT consumed in the first seven days of quitting will be compared as described for the primary outcome. Measures of abstinence will be analysed between arms as the difference in proportions with 95% confidence interval and chi-squared test.

#### *Hypothesis II: within-subgroup outcome*

Amongst those classified as not abstinent at the six month follow-up, as judged by 7-day point prevalence abstinence, an independent-samples t-test will be used to compare motivation to make another quit attempt between arms.

#### *Subgroup analyses*

The intervention effect will be estimated with 95% confidence intervals for each trial nurse separately. A formal test of whether the intervention effect varies significantly by nurse will be assessed either by using an interaction of nurse by intervention in a linear regression model, or, if the assumptions are not met, by using a chi-squared test of heterogeneity in a standard meta-analysis of the nurse intervention effects, using estimates and standard errors obtained using the bootstrap method for each nurse. For ease of interpretation, the simpler interaction test will be presented if results are not materially different. The three other subgroup variables are study centre, total daily NRT prescribed (20 mg, 27 mg, 26 mg, and 33 mg) and daily top up amount prescribed (Enhanced oral NRT, Standard oral NRT). Subgroup analyses will be confined to analysis of the primary outcome and will use the same interaction analysis framework.

#### *Handling missing data in the primary outcome*

Analysis of adherence over the 28-day period will be primarily on an intention to treat basis, involving all randomised participants. If clinic non-attenders report returning to smoking or cannot be contacted by the nurse to establish adherence, then adherence will be counted as zero for the remaining 28 day period. Missing daily adherence data from those participants who attended the clinic session will be imputed from the average taken from the days that are not missing. This analysis will be supported first by an explanatory per protocol analysis which excludes those participants not receiving the intervention, and second, by a sensitivity analysis.

### Design considerations

This is the first clinical trial evaluating the behavioural impact on adherence of prescribing medication using genetic rather than phenotypic information. There are some specific issues in choice of design for trials of interventions of this kind.

Our research questions ideally required us to randomise the information concerning the basis for prescribing (i.e. telling the two groups that their prescriptions depended on gene or nicotine dependence) and to hold the NRT treatment constant across both groups. This design was the most attractive experimentally, but unacceptable on the grounds that it would have required us to deceive participants about the true basis for their prescriptions. We considered seeking informed consent to this approach, but rejected it because of concerns that this might undermine trust in the trial as a whole.

We therefore required a design which would result in a balance of gene variants and nicotine dependence across groups and would generate similar prescription of NRT in both groups, despite the basis of prescription (genotype or phenotype) varying systematically by group. If by chance or systematic effect a greater dose of NRT was prescribed in one group than the other, then any apparent intervention effect on abstinence might be due to more effective prescribing rather than to differing expectations of effect of adherence.

We selected the design of an open-label, parallel group randomised trial in which participants were randomly allocated to one of two groups: oral NRT prescribing tailored by DNA analysis, or oral NRT prescribing tailored by nicotine dependence. We have monitored prescriptions across the groups for the need for a pre-determined change in threshold for definition of heavy smoking in the phenotype group to assure prescribing balance with the genotype group. We defined adherence to NRT rather than abstinence from smoking as our primary outcome, and adherence was defined in terms of the proportion of NRT prescribed that was consumed. This outcome is still open to the impact of differential prescribing across groups as participants might systematically consume less of a larger dose. We will analyse genotype and recorded phenotype in both trial groups thereby allowing analysis to indicate the extent to which any differences between arms could be attributed to differences in genotype or phenotype.

**Acknowledgements**
Funding
This study is funded as part of a programme of studies funded by The Medical Research Council (Grant no: G0500274: **Risk communication in preventive medicine: Optimising the impact of DNA risk information**; January 2006-January 2011, PI: Marteau).

**Author details**
[1]King's College London, Psychology Department (at Guy's), Health Psychology Section, 5th Floor Bermondsey Wing, Guy's Campus, London SE1 9RT, UK. [2]Department of Experimental Psychology, 12a Priory Road, University of Bristol, Bristol, BS8 1TU, UK. [3]Primary Care Clinical Sciences, University of Birmingham, Birmingham, B15 2TT, UK. [4]University of Oxford, Department of Clinical Pharmacology, Old Road Campus Research Building, Old Road Campus, Headington, Oxford OX3 7DQ, UK. [5]King's College London, Department of Primary Care and Public Health Sciences, 5th Floor Capital House, 42 Weston Street, London SE1 3QD, UK. [6]University of Cambridge Department of Public Health and Primary Care, Forvie Site, Robinson Way, Cambridge, CB2 0SR, UK.

**Authors' contributions**
TMM, DA, ALK, ATP, and SS are the Principal Investigators for the trial. ATP is the trial statistician, CH and TW are the trial co-ordinators, RAC, GJH and SW are the research team co-ordinators. MRM is a site lead and genetics of smoking expert. PA is a site lead and smoking cessation prescribing expert. EJ is responsible for DNA analysis. All authors drafted the manuscript and read and approved the final version. TMM is the paper guarantor.

**Competing interests**
Paul Aveyard has accepted hospitality from pharmaceutical and biotechnology companies and has been paid for work, with payments to him and his institution by such companies for work in relation to smoking cessation. The companies are Xenova Biotechnology and Pfizer.

Received: 29 September 2010 Accepted: 9 November 2010
Published: 9 November 2010

## References


1. Mooney ME, Leventhal AM, Hatsukami DK: **Attitudes and knowledge about nicotine and nicotine replacement therapy.** *Nicotine & Tobacco Research* 2006, **8**:435-446.
2. Bansal-Travers M, Cummings KM, Hyland A, Brown A, Celestino P: **Educating smokers about their cigarettes and nicotine medications.** *Health Education Research* 2010, **25**:678-686.
3. Collins F: **Has the revolution arrived?** *Nature* 2010, **464**:674-5.
4. Collins FS, Green ED, Guttmacher AE, Guyer MS: **A vision for the future of genomics research: A blueprint for the genomic era.** *Nature* 2003, **422(6934)**:835-847.
5. Gramling R, Nash J, Siren K, Culpepper L: **Predictive genetics in primary care: expectations for the motivational impact of genetic testing affects the importance family physicians place on screening for familial cancer risk.** *Genetics in Medicine* 2003, **5**:172-175.
6. Chen S, Chaiken S: **The heuristic-systematic model in its broader context.** In *Dual process theories in social psychology.* Edited by: Chaiken S, Trope Y. New York: Guildford Press; 1999:73-96.
7. Li MD: **The genetics of nicotine dependence.** *Curr Psychiatry Rep* 2006, **8**:158-164.
8. Li MD, Cheng R, Ma JZ, Swan GE: **A meta-analysis of estimated genetic and environmental effects on smoking behavior in male and female adult twins.** *Addiction* 2003, **98**:23-31.
9. Schnoll RA, Johnson TA, Lerman C: **Genetics and smoking behavior.** *Curr Psychiatry Rep* 2007, **9**:349-357.
10. Lerman C, Schnoll RA, Munafò MR: **Genetics and smoking cessation: Improving outcome in patients at risk.** *American Journal of Preventive Medicine* 2007, **33**:S398-S405.
11. Munafò MR, Lerman C, Niaura R, Shields AE, Swan GE: **Smoking cessation treatment: Pharmacogenetic assessment.** *Current Opinion in Molecular Therapeutics* 2005, **7**:202-208.
12. Munafò MR, Shields AE, Berrettini WH, Patterson F, Lerman C: **Pharmacogenetics and nicotine addiction treatment.** *Pharmacogenomics* 2005, **6**:211-223.
13. Zhang Y, Wang D, Johnson AD, Papp AC, Sadee W: **Allelic expression imbalance of human mu opioid receptor (OPRM1) caused by variant A118G.** *J Biol Chem* 2005, **280**:32618-32624.
14. Bond C, LaForge KS, Tian M, Melia D, Zhang S, Borg L, Gong J, Schluger J, Strong JA, Leal SM, Tischfield JA, Kreek MJ, Yu L: **Single nucleotide polymorphism in the human mu-opioid receptor gene alters beta-endorphin binding and activity: possible implications for opiate addiction.** *Proc Natl Acad Sci USA* 1998, **95**:9608-9613.
15. Befort K, Filliol D, Decaillot FM, Gaveriaux-Ruff C, Hoehe MR, Kieffer BL: **A single nucleotide polymorphic mutation in the human mu-opioid receptor severely impairs receptor signaling.** *J Biol Chem* 2001, **276**:3130-3137.
16. Beyer A, Koch T, Schroder H, Schulz S, Hollt V: **Effect of the A118G polymorphism on binding affinity, potency and agonist-mediated endocytosis, desensitization, and resensitization of the human muopioid receptor.** *J Neurochem* 2004, **89**:553-560.
17. Lerman C, Wileyto EP, Patterson F, Rukstalis M, Audrain-McGovern J, Restine S, Shields PG, Kaufmann V, Redden D, Benowitz N, Berrettini WH: **The functional mu opioid receptor (*OPRM1*) Asn40Asp variant predicts short-term response to nicotine replacement therapy in a clinical trial.** *Pharmacogenomics Journal* 2004, **4**:184-192.
18. Munafò MR, Elliot KM, Murphy MFG, Walton RT, Johnstone EC: **Association of the mu-opioid receptor gene with smoking cessation.** *The Pharmacogenomics Journal* 2007, **7**:353-361.
19. Marteau TM, Weinman J: **Self-regulation and the behavioural response to DNA risk information: A theoretical analysis and framework for future research.** *Social Science & Medicine* 2006, **62(6)**:1360-1368.
20. Iselin MG, Addis ME: **Effects of etiology on perceived helpfulness of treatments for depression.** *Cognitive therapy and research* 2003, **27**:205-222.
21. Marteau T, Senior V, Humphries SE, Bobrow M, Cranston T, Crook MA, Day L, Fernandez M, Horne R, Iversen A, Jackson Z, Lynas J, Middleton-Price H, Savine R, Sikorski J, Watson M, Weinman, J, Wierzbicki AS, Wray R: **Psychological impact of genetic testing for familial hypercholesterolemia within a previously aware population: A randomized controlled trial.** *American Journal of Medical Genetics* 2004, **128A**:285-293.
22. Phelan JC, Yang LH, Cruz-Rojas R: **Effects of attributing serious mental illnesses to genetic causes on orientations to treatment.** *Psychiatric Services* 2006, **57**:382-387.
23. Wright AJ, Weinman J, Marteau TM: **The impact of learning of a genetic predisposition to nicotine dependence: an analogue study.** *Tobacco Control* 2003, **12**:227-230.
24. McBride CM, Bepler G, Lipkus IM, Lyna P, Samsa G, Albright J, Datta S, Rimer BK: **Incorporating genetic susceptibility feedback into a smoking cessation program for African-American smokers with low income.** *Cancer Epidemiology, Biomarkers & Prevention* 2002, **11**:521-528.
25. Wu JR, Moser DK, Lennie TA, Burkhart PV: **Medication adherence in patients who have heart failure: A review of the literature.** *Nursing Clinics of North America* 2008, **43**:133-153.
26. Shiffman S: **Use of more nicotine lozenges leads to better success in quitting smoking.** *Addiction* 2007, **102**:809-814.
27. Shiffman S: **Effect of nicotine lozenges on affective smoking withdrawal symptoms: Secondary analysis of a randomized, double-blind, placebo-controlled clinical trial.** *Clinical Therapeutics* 2008, **30(8)**:1461-1475.
28. **Information Centre for Health and Social Care (2008) Statistics on NHS Stop smoking Services in England April 2007-December 2007.** [http://www.ic.nhs.uk/statistics-and-data-collections/health-and-lifestyles/nhs-stop-smoking-services/statistics-on-nhs-stop-smoking-services-in-england-april-2007–december-2007-q3–quarterly-report], (Retrieved September 21st 2009).
29. Shiloh S, Rashuk-Rosenthal D, Benyamini Y: **Illness causal attributions: an exploratory study of their structure and associations with other illness cognitions and perceptions of control.** *J Behav Med* 2002, **25**:373-394.
30. Miller SA, Dykes DD, Potesky HF: **A simple salting out procedure for extracting DNA from human nucleated cells.** *Nucleic Acids Research* 1988, **6(3)**:1215.
31. Aveyard P, West R: **Managing smoking cessation.** *British Medical Journal* 2007, **335(7609)**:37-41.
32. Silagy C, Lancaster T, Stead L, Mant D, Fowler G: **Nicotine replacement therapy for smoking cessation.** *Cochrane Database of Systematic Reviews* 2004, , **3**: CD000146.
33. Stead LF, Perera R, Bullen C, Mant D, Lancaster T: **Nicotine replacement therapy for smoking cessation (Review).** *Cochrane Database of Systematic Reviews* 2008, **1**.
34. Hajek P: **Withdrawal-oriented Therapy for Smokers.** *British Journal of Addiction* 1989, **84**:591-598.
35. NCSCT (NHS Centre for Smoking Cessation and Training): **Learning Outcomes for Training Stop Smoking Specialists.** London: NCSCT; 2010.
36. Heatherton TF, Kozlowski LT, Frecker RC, Fagerstrom KO: **The Fagerstrom Test for Nicotine Dependence: a revision of the Fagerstrom Tolerance Questionnaire.** *British Journal of Addiction* 1991, **86**:119-127.
37. Aveyard P, Brown K, Saunders C, Alexander A, Johnstone E, Munafò MR, Murphy M: **Weekly versus basic smoking cessation support in primary care: a randomised controlled trial.** *Thorax* 2007, **62**:898-903.
38. National Institute for Health and Clinical Excellence: *Guidance on the use of nicotine replacement therapy (NRT) and bupropion for smoking cessation* 2002 [http://www.nice.org.uk/cat.asp?c=30590], Retrieved September 08, 2008.
39. Hughes JR, Keely JP, Niaura RS, Ossip-Klein DJ, Richmond RL, Swan GE: **Measures of abstinence in clinical trials: issues and recommendations.** *Nicotine &Tobacco Research* 2003, **5**:13-25.
40. West R, Hajek P, Stead L, Stapleton J: **Outcome criteria in smoking cessation trials: proposal for a common standard.** *Addiction* 2005, **100**:299-303.
41. Alterman AI, Gariti P, Cook TG, Cnaan A: **Nicodermal patch adherence and its correlates.** *Drug & alcohol dependence* 1999, **53**:159-165.


### Pre-publication history

The pre-publication history for this paper can be accessed here:
http://www.biomedcentral.com/1471-2458/10/680/prepub

doi:10.1186/1471-2458-10-680
**Cite this article as:** Marteau *et al.*: Trial Protocol: Using genotype to tailor prescribing of nicotine replacement therapy: a randomised controlled trial assessing impact of communication upon adherence. *BMC Public Health* 2010 **10**:680.
